# Supplementary figures and images for: Resistance training prevents right ventricle hypertrophy in rats exposed to secondhand cigarette smoke
Source: PLoS One. 2020 Aug 7;15(8):e0236988. doi: 10.1371/journal.pone.0236988 (PMC7413484; doi:10.1371/journal.pone.0236988)

## Slide 1
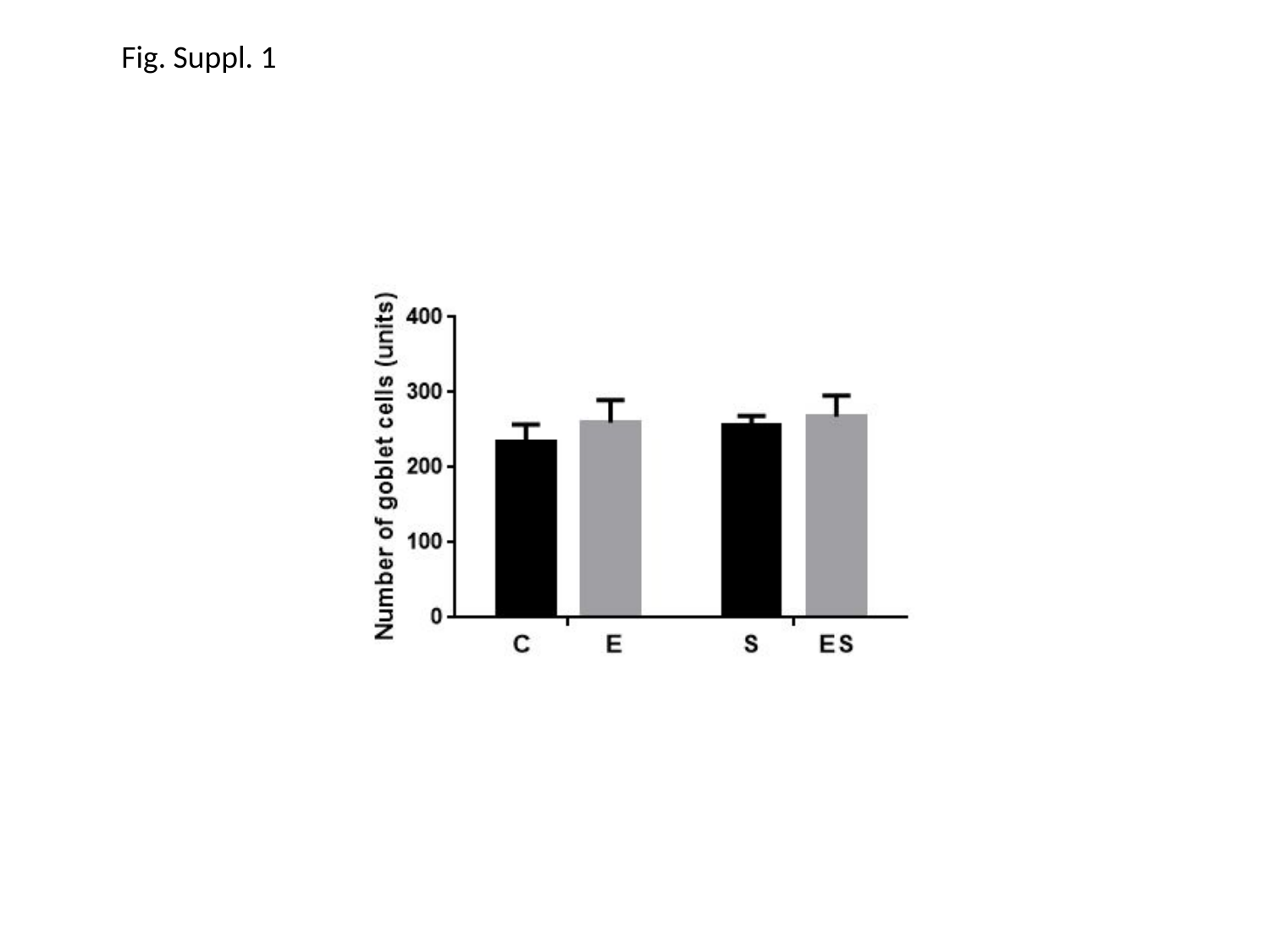

Fig. Suppl. 1

Supplement: S1 Fig — Values of the sum of 10 fields expressed as the mean ± SEM (n = 8 / group). (PPTX) [file pone.0236988.s001.pptx]

## Slide 1
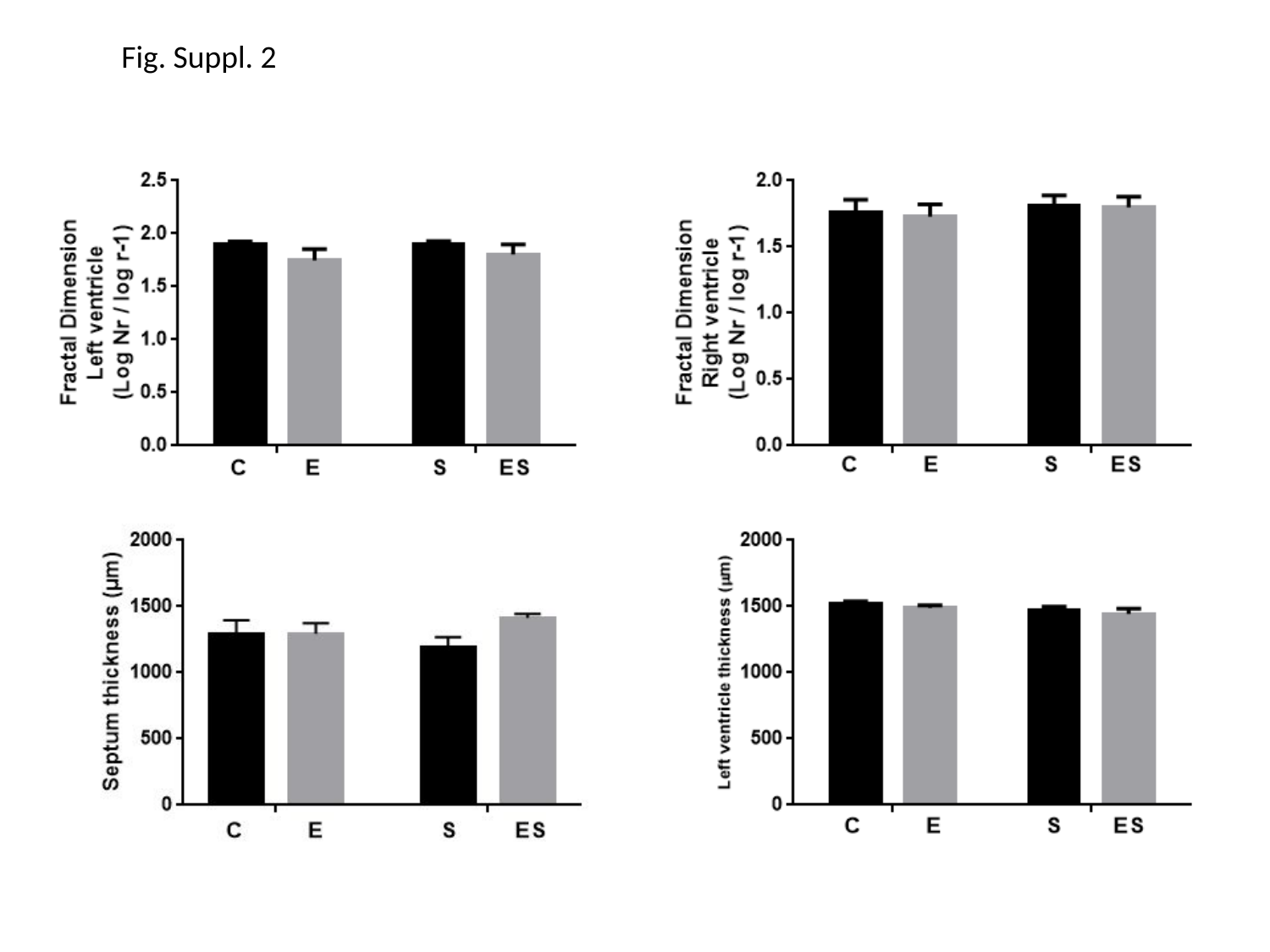

Fig. Suppl. 2

Supplement: S2 Fig — In A, thickness of the left ventricle (in μm). In B, thickness of the septum (in μm). In C, fractal dimension of the left ventricle (Log Nr / log r-1). In D, fractal dimension of the right ventricle (Log Nr / log r-1). No significant alteration was observed. Values expressed as the mean ± SEM (n = 8 / group). (PPTX) [file pone.0236988.s002.pptx]
