# Supplementary material for: Resistance training prevents right ventricle hypertrophy in rats exposed to secondhand cigarette smoke
Source: PLoS One. 2020 Aug 7;15(8):e0236988. doi: 10.1371/journal.pone.0236988 (PMC7413484; doi:10.1371/journal.pone.0236988)
Supplement: S1 Table — (DOCX) [file pone.0236988.s003.docx]

Suppl.Table 1 - MSL weight - Details exercise capacity and MSL weight for E and ES groups in the beginning of the resistance training protocol.

| Load  Group | 50% BW | 75% BW | 90% BW | 100% BW |
| --- | --- | --- | --- | --- |
| E | 130.87±6.76 | 196.37±10.19 | 235.68±12.23 | 261.87±13.59 |
| ES | 117.68±6.80 | 212.17±10.20 | 235.75±12.25 | 235.75±13.51 |

Values ​​expressed as the mean ± SEM (n = 8 / group). BW = body weight
