# Supplementary material for: Resistance training prevents right ventricle hypertrophy in rats exposed to secondhand cigarette smoke
Source: PLoS One. 2020 Aug 7;15(8):e0236988. doi: 10.1371/journal.pone.0236988 (PMC7413484; doi:10.1371/journal.pone.0236988)
Supplement: S1 File — (DOCX) [file pone.0236988.s004.docx]

| Title | 1 | Resistance training prevents right ventricle hypertrophy in rats exposed to secondhand cigarette smoke |
| --- | --- | --- |
| Abstract | 2 | Exposure to secondhand cigarette smoke is associated with the development of diverse diseases. Resistance training has been considered one of the most useful tools for patients with pulmonary disease, improving their quality of life. This study aimed to evaluate the effect of resistance training (RT) on the prevention of thickening of the right ventricle wall of rats exposed to secondhand cigarette smoke. Thirty-two male Wistar rats aged 2-month-old were divided into four groups: Control (C), Smoker (S), Exercised (E) and Exercised Smoker (ES). The smoker groups were exposed to the smoke of four cigarettes for 30 min, twice daily, five days a week, for 16 weeks. The exercised groups climbed on a vertical ladder with progressive load, once a day, five days a week, for 16 weeks. The heart, trachea, lung, liver and gastrocnemius muscle were removed for histopathological analysis. Pulmonary emphysema (S and ES *vs* C and E, *P* <0.0001) and pulmonary artery thickness enlargement (S *vs* C and E, *P* = 0.003, ES *vs* C, *P* = 0.003) were detected in the smoking groups. There was an increase in the right ventricle thickness in the S group compared with all other groups (*P* <0.0001). An increase in resident macrophages in the liver was detected in both smoking groups compared with the C group (*P* = 0.002). Additionally, a relevant reduction of the diameter of the muscle fibers was detected only in ES compared with the C, S and E groups (*P* = 0.0002), impairing, at least in part, the muscle mass in exercised smoking rats. Therefore, it was concluded that resistance training prevented the increase of thickness of the right ventricle in rats exposed to secondhand cigarette smoke, but it may be not so beneficial for the skeletal muscle of smoking rats. |
| Background | 3 | Smoking is known to be a chronic and epidemic disease and is predicted to cause more than 10 million deaths by 2030 (Zatonski et al., 2018). Secondhand smoke is the combination of two smokes: the mainstream exhaled by smokers plus the burning end of a cigarette (sidestream). Its smoke contains many noxious substances, with hundreds toxic, and some can trigger câncer (US Department of Health and Human Services,2014). Exposure to secondhand cigarette smoke is related to the development of more than 50 diseases, including pulmonary emphysema and chronic bronchitis, which compose chronic obstructive pulmonary disease (COPD) (US Department of Health and Human Services,2014). Recently, exercise in the form of resistance training (RT) was classified as one of the best and most useful options for the treatment of patients with pulmonary diseases (Gosselin et al., 2003; Ryrso et al., 2018).  Kozma et al. (2014) concluded that exposure to secondary cigarette smoke is capable of developing a model of oxidative lung injury and inflammation, accelerating functional and morphological alterations, and limiting gas exchange. However, this type of exposure has not yet been associated with resistance training, which has also been investigated as a tool similar to strength training in humans (Hornberger et al. ,2004, Leite et al., 2013), enabling load readjustment according to the strength of each animal (Lima et al., 2019; Johnson et al., 2000).  Although several benefits of RT have already been described in the literature, there is no consensus about its real contribution to the prevention of tissue alterations provoked by exposure to secondhand cigarette smoke. The present study aimed to evaluate the effect of resistance training on anatomopathological changes in the right and left ventricles, trachea, lung, gastrocnemius muscle and liver of rats exposed to secondhand smoke.  References:   1. Gosselin N, Lambert K, Poulain M, Martin A, Prefaut C, Varray A. Endurance training improves skeletal muscle electrical activity inactive copd patients. Muscle & Nerve. 2003; 28(6),744-753. 2. Hornberger TA, Farrar RP. Physiological hypertrophy of the FHL muscle following 8 weeks of progressive resistance exercise in the rat. Can J Appl Physiol. 2004;29(1),16-31. 3. Johnson JW. A heuristic method for estimating the relative weight of predictor variables in multiple regression. Multivariate Behav Res. 2000;35(1):1-19. 4. Kozma RDLH, Alves EM, Barbosa OVA, Lopes FDTQD, Guardia RC, Buzo HV, et al. A new experimental model of cigarette smoke-induced emphysema in Wistar rats. J Bras Pneumol. 2014;40(1):46-54. 5. Leite RD, Durigan RC, de Souza LAD, de Souza CMV, Souza Md, Selistre-de-Araújo HS, et al. Resistance training may concomitantly benefit body composition, blood pressure and muscle MMP-2 activity on the left ventricle of high-fat fed diet rats. Metabolism. 2013;62(10):1477-1484. 6. Lima YC, Kurauti MA, da Fonseca Alves G, Ferezini J, Piovan S, Malta A, et al. Whey protein sweetened with Stevia rebaudiana Bertoni (Bert.) increases mitochondrial biogenesis markers in the skeletal muscle of resistance-trained rats. Nutr Metab (Lond). 2019;16(1):1-11. 7. Ryrso CK, Thaning P, Siebenmann C, Lundby C, Lange P, Pedersen BK, et al. Effect of endurance versus resistance training on local muscle and systemic inflammation and oxidative stress in COPD. Scand J Med Sci Sports. 2018, 8(11):2339-2348. 8. US Department of Health and Human Services. Let's make the next generation tobacco-free: your guide to the 50th anniversary Surgeon General's report on smoking and health. Atlanta: US Department of Health and Human Services, Centers for Disease Control and Prevention. National Center for Chronic Disease Prevention and Health Promotion, Office on Smoking and Health, 2014. 9. Zatonski WA. World No Tobacco Day “Tobacco and heart disease”. J Health Inequal. 2018;4(1):18-18.   b. Explain how and why the animal species and model being used can address the scientific objectives and, where appropriate, the study’s relevance to human biology.  Wistar rats were used as animal model for our study since they present many similarity with humans considering physiologycal functions and they belong to a strain widely used in scientific studies. Also, as we performed the evaluation in different organs such as heart, lungs, liver, skeletal muscle, we really needed to use animal model. Another important point is that the study could not be carried out in vitro or in silico since we want to investigate the effect of resistance training considering its action on the body as a whole. We chose to use male rats because of fewer hormone interference compared to female rats, besides many studies in the literature use male rats as animal model. |
| Objectives | 4 | The present study aimed to evaluate the effect of resistance training on anatomopathological changes in the right and left ventricles, trachea, lung, gastrocnemius muscle and liver of rats exposed to secondhand smoke.  The hypothesis is that resistance training would be beneficial to smoking rats, preventing possible anatomopathological changes provoked by secondhand cigarette smoke exposure. |
| Ethical statement | 5 | All procedures complied with the ethical principles of animal research and were approved by the Ethical Committee for Animal Research of the School of Sciences and Technology, Sao Paulo State University, Presidente Prudente (# 02/2017). |
| Study design | 6 | Thirty-two male Wistar rats, aged 45 days, were separated into cages with four animals per cage and kept under a controlled temperature (23 ± 2ºC), in a light/dark cycle (12 h/12 h) room, with free access to water. The cages were filled with environmental enrichment, and all procedures were carefully done to reduce stress level, and ensure the welfare of the animals. They spent 14 days in this room without any intervention corresponding to adaptation period.  Every day the animals were monitoring by observation of behavior and intake food and water, and no alteration was noticed during all procedures with the animals.  Forty grams of commercial chow (Supralab - Alisul, Maringa, PR, Brazil) were offerred to each rat, every Monday, Wednesday and Friday. The rest of the chow was weighed and subtracted from the initial offer (40 g) for monitoring.  The weight gain was determined by subtracting the initial weight from the final weight on the euthanasia day.  After the adaptation period, resistance training (RT) and exposure to secondhand cigarette smoke (SCS) were performed for 16 weeks.  The animals was randomly divided into four groups: Control (C / n = 08) - without interventions; Exercised (E / n = 08) - performed RT; Smoker (S / n = 08) exposed to SCS; Exercised Smoker (ES / n = 08) - exposed to SCS and performed RT.  The exercised groups (E and ES) performed the climbing protocol previously reported in the literature by Hornberger and Farrar (2004), which mimics progressive resistance exercise in humans. Two phases were determined: adaptation and experimental. During the experimental phase, the researchers were not blinded and the cages were identified with the corresponding group.  The adaptation phasis comprised the first 4 days of the intervention, in which the animals adapted to the climbing exercise by being stimulated by a manual stimulus in their rostral portion to climb to a cage present at the top of a ladder, where they rested for 60 seconds. The protocol was repeated four times a day.  On the fifth day, the rats performed the maximum supported load (MSL) test to determine which initial load could be supported by each animal (Leite et al. 2013; Lima et al. 2019). A glove containing washers inside was used as the load, and it was fixed to the tail of the animal by adhesive tape. The initial load was fixed at 75% of body weight for each animal. After each complete climb, 30 grams of weight was added. The test was interrupted if the animal performed more than eight repetitions and if, after three consecutive stimuli, the animal could not perform the climb. The maximum load recorded was the weight corresponding to the last complete climb.  The training consisted of four series of climbs on a ladder, with intervals of 60 seconds, five times a week (on consecutive days), once per day. The training started with 50% of 01 MSL for each animal with the load increasing to 75%, 90% and 100% of 01 MSL every 4 weeks, resulting in an experimental period with 16 weeks.  The smoking rats S and ES were exposed to secondhand cigarette smoke. In the first week (adaptation phasis), 04 animals in both groups, S and ES, were exposed to secondhand cigarette smoke from combustion of only 2 cigarettes for 10 min, once daily. After this period, 04 smoking rats of each group (S or ES) were exposed to secondhand smoke from the combustion of 04 cigarettes for 30 min, 2 times a day, 5 days / week, for 16 weeks. This dose, which totaled 8 cigarettes / day, was determined from previous studies that reported this amount being the dose tolerated by the animals, while avoiding the risk of mortality Ebersbach Silva P, 2013; Kamiide Y, 2015).  After 16 weeks of interventions, euthanasia occurred after anesthesia with intraperitoneal injection of ketamine hydrochloride (60 mg / kg body weight) and xylazine hydrochloride (10 mg / kg body weight) after 12 h of fasting, 24 h after the last RT and SCS exposure for removal of organs (liver, trachea, lung, heart and gastrocnemius skeletal muscle). Euthanasia occurred by exsanguination.  All histopathological analysis was blinded. The slides were identified with the information of the corresponding animal, however this information was covered with tape during photos and analysis. A second researcher was responsible for quantifying the information analyzed and separating it into their respective groups for future statistical analysis.  References:  1- Hornberger TA, Farrar RP. Physiological hypertrophy of the FHL muscle following 8 weeks of progressive resistance exercise in the rat. Can J Appl Physiol. 2004;29(1),16-31.  2- Leite RD, Durigan RC, de Souza LAD, de Souza CMV, Souza Md, Selistre-de-Araújo HS, et al. Resistance training may concomitantly benefit body composition, blood pressure and muscle MMP-2 activity on the left ventricle of high-fat fed diet rats. Metabolism. 2013;62(10):1477-1484.  3- Lima YC, Kurauti MA, da Fonseca Alves G, Ferezini J, Piovan S, Malta A, et al. Whey protein sweetened with Stevia rebaudiana Bertoni (Bert.) increases mitochondrial biogenesis markers in the skeletal muscle of resistance-trained rats. Nutr Metab (Lond). 2019;16(1):1-11.  4- Ebersbach Silva P, Alves T, Fonseca ATS, Oliveira MADN, Machado UF, et al. Cigarette smoke exposure severely reduces peripheral insulin sensitivity without changing GLUT4 expression in oxidative muscle of Wistar rats. Arq Bras Endocrinol Metabol. 2013;57(1):19-26.  5- Kamiide Y, Furuya M, Inomata N, Yada T. Chronic exposure to cigarette smoke causes extrapulmonary abnormalities in rats. Environ Toxicol Pharmacol. 2015;39(2):864-870.  Timeline of interventions:  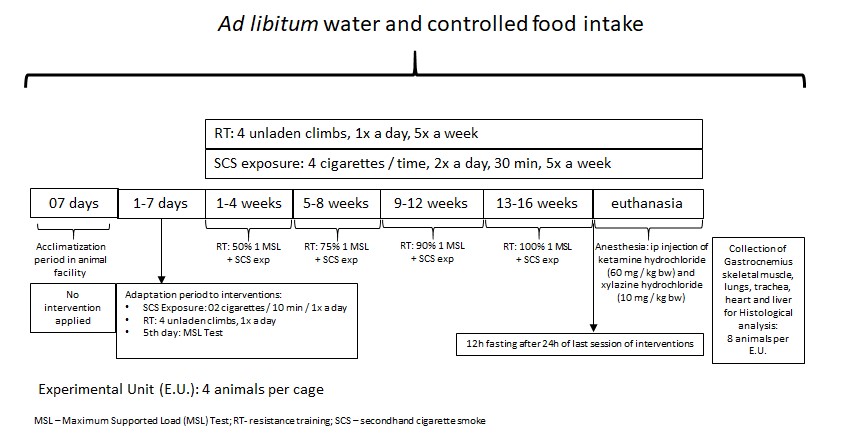 |
| Experimental procedures | 7 | Two interventions directly involving animals were performed: secondhand cigarette smoke (SCS) exposure and resistance training (RT).  - SCS exposure:  The inhalation system was a custom built system composed by a closed glass box (100 x 44 x 44 cm), divided in 2 different compartments: one compartiment for allocation of the cage with 04 rats to be exposed to the cigarette smoke, and the other for the allocation of the cigarettes to be burned. A compressor of 10 L/min-air was coupled to the cigarette compartment to push the smoke to the other compartment of the box. The compartment where the animal was allocated presented a hole for the exhaust of the smoke. Four cigarettes were lit and the complete combustion occurred during 10 minutes, however the rats spent 30 minutes inside this system. So, during the 10 first minutes inside the chamber, the cigarettes were burned, but in the rest of the time the rats were exposed only to the polluted air of the environment. This dose of smoke exposure is equivalent to 10 to 20 cigarettes for a chronic human smoker. Commercial cigarettes (Malboro Red®, Philip Morris International, Brazil) containing 10mg of tar, 0.8 mg of nicotine, and 10 mg of carbon monoxide during the combustion were used in this study, with the same components as found in the literature.  The smoking rats (S and ES) were exposed to secondhand cigarette. In the first week, 04 animals in the S and ES groups were exposed to secondhand cigarette smoke from combustion of only 2 cigarettes for 10 min, once daily. After this period, 04 smoking rats (S or ES) were exposed to secondhand smoke from the combustion of 04 cigarettes for 30 min, 2 times a day, 5 days / week, for 16 weeks. This dose, which totaled 8 cigarettes / day, was determined from previous studies that reported this amount being the dose tolerated by the animals, while avoiding the risk of mortality.  The exposure has always ocurred in the morning (10:00 a.m. and 2:00 p.m.).  - RT  The resistance training (RT) was performed using a vertical ladder (80 ° inclination), and a progressive load inside gloves that were attached to the animal's tail. The adaptation phasis comprised the first 4 days of the intervention, in which the animals adapted to the climbing exercise by being stimulated by a manual stimulus in their rostral portion to climb to a cage present at the top of a ladder, where they were able to rest for 60 seconds. The protocol was repeated four times a day.  On the fifth day, the rats performed the maximum supported load test to determine which initial load could be supported by each animal (Leite RD, 2013; Lima YC, 2019). A glove containing washers inside was used as the load, and it was fixed to the tail of the animal by adhesive tape. The initial load was fixed at 75% of body weight for each animal. After each complete climb, 30 grams of weight was added. The test was interrupted if the animal performed more than eight repetitions and if, after three consecutive stimuli, the animal could not perform the climb. The maximum load recorded was the weight corresponding to the last complete climb.  The training consisted of four series of climbs on a ladder, with intervals of 60 seconds, five times a week (on consecutive days), once per day. The training started with 50% of 01 MSL for each animal with the load increasing to 75%, 90% and 100% of 01 MSL every 4 weeks, resulting in an experimental period with 16 weeks of intervention, characteristic of long-term exercise.  The training was always performed in the morning before smoking (9:00 a.m.).  Anesthesia with intraperitoneal ketamine hydrochloride (60 mg / kg body weight) and xylazine hydrochloride (10 mg / kg body weight) was injected after 12 h of fasting, 24 h after the last RT and smoking sessions for removal of organs (liver, trachea, lung, heart and gastrocnemius skeletal muscle) for histological analysis and weighing. Euthanasia occurred by exsanguination. Naso-anal length was measured with a ruler and body mass was weighed in the scale for calculation of Lee Index (weight^1/3^/naso-anal length). |
| Experimental animals | 8 | Thirty-two male Wistar rats (Rattus Norvegicus), aged 45 days, weighing around 250g, were randomly divided by lottery into 04 groups, and 04 rats per cage. These animals were obtained from the Central Animal Laboratory of UNESP, campus Botucatu, SP, Brazil, and kept into a room with controlled luminosity (light-dark cycle, 12h/12h), temperature (23+/-2ºC) and umidity in vivarium at UNESP, campus Presidente Prudente, SP, Brazil. The four experimental groups were: C – control rats without interventions; E – exercised rats who performed resistance training (RT) during 16 weeks; S – smoking rats who were submitted to secondhand cigarette smoke (SCS) exposure during 16 weeks; SE – smoking and exercised rats – who performed RT and were submitted to SCS exposure during 16 weeks. |
| Housing and husbandry | 9 | The rats were allocated in collective solid-bottomed polyethylene cages model, with dimensions of 41 x 34 x 18 cm, containing 4 animals per cage, and so, 02 cages of each group. The bed provided was pine shavings sawdust, briefly autoclaved to ensure the absence of pathogens. This bed was changed every 2 days. We always respected the relay scheme of cages for changing and cleaning. We began with the first cage of C group and after the second cage of C group, followed by the first cage of E group, and the second cage of E group, and so on. Next day of cleaning and change, we began with the E group, followed by S group, ES group, and C group. Next day of cleaning and change, we began with cages of E group, followed by S, C and S groups. Next change the sequence was ES, C, S, E. And so on.  The animals were kept in a private masonry room, with an exhaust fan and controlled temperature (23 ± 2ºC), without windows and with antiport to guarantee an effective light and dark cycle. The lights were turned on at 7:00 am and turned off at 7:00 pm (12 h / 12 h).  These animals received rolls of paper daily as environmental enrichment to gnaw and relax. Every day the animals were monitoring by observation of behavior and intake food and water, and no alteration was noticed during all procedures with the animals.  Commercial standard chow and water were freely offerred during all time of life of the animals. We control the intake of food as a way to verify the wellness of the animals. So, forty grams of commercial chow (Supralab - Alisul, Maringa, PR, Brazil) were offered to each rat (160g per cage), every Monday, Wednesday and Friday. The rest of the chow was weighed and subtracted from the initial offer (40 g) for monitoring. |
| Sample size | 10 | Thirty-two male Wistar rats, were divided into four groups: Control (C / n = 08) - without interventions; Exercised (E / n = 08) - performed RT; Smoker (S / n = 08) exposed to secondhand cigarette smoke; Exercised Smoker (ES / n = 08) - exposed to secondhand smoke exposure and performed RT.  A statistical power analysis tool from the PSS Calculations version 3.1.2 program was used to determine the number of animals per test, which has already been shown to be effective in previous tests carried out in the group (analysis criteria: 80% of statistical power , alpha: 0.05, expected significant difference between averages of 30% and expected standard deviation of 20%), which gave us the confidence that we would use the smallest possible number of animals to obtain statistically significant data.  The number of animals determined by the statistical power analysis was 08 per group. |
| Allocating animals  to experimental groups | 11 | Thirty-two male Wistar rats, aged 45 days, were separated into cages with four animals per cage and kept under a controlled temperature (23 ± 2ºC), in a light/dark cycle (12 h/12 h) room, with free access to water. The cages were filled with environmental enrichment (paper roll).  The animals were randomly divided by lottery into four groups: Control (C / n = 08) - without interventions; Exercised (E / n = 08) - performed Resistance Training; Smoker (S / n = 08) - exposed to secondhand cigarette smoke; Exercised Smoker (ES / n = 08) - exposed to secondhand cigarette smoke and performed Resistance Training.  Four animals were allocated per cage, so we had two boxes for each group, totaling 8 boxes in this study.  The intervention occurred by switched boxes. The training was performed from 9:00 a.m., with a relay scheme, starting with the first box of the group E performing training, followed by the first in the group ES. After, the second box of the E group was followed by the second of the ES group and so on until the fourth box of each exercised group. On the second day, the first box of the E group to train was the second box of the E group followed by the second box of the ES group, and after, the third box of each group, the fourth box of each group, and the first box othe the day 1, was the last box to train on this day. And so on, always switching the sequence of the boxes.  The same was done for the secondhand cigarette smoke exposure. All groups were exposed to cigarette smoke at 9:00 am and 2:00 pm. The first day began with the first box of the ES group followed by the first box of the S group. After, the second box of the SE group foloowed by the second box of the S group, and soo n until the fourth box of each smoking group. On the second day, the first box of the ES group was the last to be exposed, and so on.  ES group always performed RT before the exposure to the cigarette smoke.  Control group was always shifted to the experimental room to simulate the same situation of locomotion of the boxes, and after they were returned to the animals room. |
| Experimental outcomes | 12 | There was no intercorrence during the experimental procedures.  No sample was excluded from the statistics, with no outliers, assessed by GraphPad QuickCalcs.  During 07 days of acclimatization in the new environment, and during the adaptation period to the interventions, no behavioral change was detected in the animals. Food and water were offerred ad libitum. We evaluted weight every week and all animals presented increase of weight. They were allocated in four per cage to maintain the sociability and to avoid distress.  During the MSL test, we performed the test separately for each animal per time.  During the secondhand cigarette smoke exposure, four animals per cage were submitted to the exposure per time. S and ES groups were exposed to secondhand smoke from the combustion of 04 cigarettes for 30 min, 2 times a day, 5 days / week, for 16 weeks with 350 ppm CO / exposure, measured by specific gas detector (ToxiPro® da Biosystems) placed inside the chamber.  The training consisted of four series of climbs on a ladder, with intervals of 60 seconds, five times a week (on consecutive days), once per day. The training started with 50% of 01 MSL for each animal with the load increasing to 75%, 90% and 100% of 01 MSL every 4 weeks, resulting in an experimental period with 16 weeks of intervention.  The training for smoking group was performed previously the cigarette smoke exposure.  We always respected the relay scheme of boxes for both interventions: training and smoke exposure.  During the period of interventions, food was controlled by offer of 160 grams of standard chow per cage. The rest of chow in the cage was measured and subtracted from the initial offer for monitoring.  Anesthesia with intraperitoneal injection of ketamine hydrochloride (60 mg / kg body weight) and xylazine hydrochloride (10 mg / kg body weight) was injected after 12 h of fasting, 24 h after the last RT and smoking sessions for removal of organs (liver, trachea, lung, heart and gastrocnemius skeletal muscle) for histological analysis and weighing. Euthanasia occurred by exsanguination.  Naso-anal length was measured with a ruler and body mass was weighed in the scale for calculation of Lee Index (weight^1/3^/naso-anal length).  Organs (liver, trachea, heart, gastrocnemius skeletal muscle, lungs) were removed for histological analysis and weight. |
| Statistical methods | 13 | **Body mass gain** - (initial weight - final weight), ANOVA-Two-way- Tukey's.  **Final body weight** - final weight, ANOVA-Two-way- Tukey's.  **Food** **consumption** - Animal food intake was checked three times a week. These 3 values were added (to have a weekly consumption idea), divided by 7 (to have a daily consumption) and again divided by 4 (due to the number of animals in each box, to have a daily consumption per animal). ANOVA-Two-way- Tukey's  **Lee Index** - (weight ^1/3^/naso-anal length in cm), ANOVA-Two-way- Tukey's.  **Skeletal muscle gastrocnemius** - weight of the right paw muscle, collected after euthanasia and weighed on a high precision scale. ANOVA-Two-way- Tukey's  **Goblet cell counts** - 10 photos corresponding to approximately 1 mm² were taken to identify the number of goblet cells in each trachea. The value of each animal was added up and used for statistics. ANOVA-Two-way- Tukey's.  **Pulmonary emphysema** - Pulmonary emphysema was diagnosed when the alveolar spaces were enlarged and the alveolar septa retracted. All S animals and 7 SE animals presented pulmonary emphysema. Kruskal-Wallis.  **Pulmonary artery thickness** - For analysis of pulmonary artery thickness was Alcian Blue-PAS staining, with two photos per animal and two measurements per photo (magnification of 200x). The mean of each photo was used for statistical analysis. ANOVA-Two-way- Tukey's.  **Ventricle thickness (right, left and septum)** - HE staining to measure the thickness of the left and right ventricular free walls and the interventricular septum. One photo of each area (magnification of 100x) was taken with two measurements per picture. The mean of each photo was used for statistical analysis. ANOVA-Two-way- Tukey's.  **Diameter of the fibers of the gastrocnemius muscle** - HE staining was used in the gastrocnemius skeletal muscle. Sections were taken from the central area of the muscle. The analysis of the diameter of the muscle fibers was performed in 50 intact fibers in the HPF photos. Statistics were performed with the average of 50 measurements for each animal. ANOVA-Two-way- Tukey's.  **Kupffer cell counting** - The Kupffer cells (resident macrophages) were counted in 10 HPF, corresponding to approximately 1 mm² in each sample. The statistics were performed with the sum of the 10 fields of each animal. ANOVA-Two-way- Tukey's.  **Collagen Density (Fractal Dimension) -** To perform the analysis of the fractal dimension of the right and left ventricles, the slides stained with Masson's trichrome were photographed (one photo for each area for each animal) and passed through the binarization process to read and analyze the fractal dimension with the box-counting method, using free ImageJ (NIH) software ([http://rsbweb.nih.gov/ij/](about:blank)). ImageJ software performs box counting in two dimensions, allowing the quantification of the distribution of pixels in the space. The fractal analysis of the histological slides is the relation between the resolution and the evaluated scale: DF = (Log Nr / log r-1), with Nr being the amount of equal elements needed to fill the original object and r being the scale applied to the object. ANOVA-Two-way- Tukey's. |
| Baseline data | 14 | No death occurred during the experiment. Any relevant health changewas detected along the experiment.  All groups showed similar weight in the beginning of the experiment: C=252.12 ± 10.2; E= 246.67 ± 12.6; S=250.42 ± 8.7; ES=244.91 ± 12.5. At the end of experiment ES showed significant reduction compared to C rats, accompanied by reduced chow intake, as discussed in the study. Final weight: C = 482.62 ± 12.71, E = 463.87 ± 15.30, S = 435.12 ± 6.80 and ES = 414.01 ± 16.04*, *P*<0.001 vs C. Chow intake: C = 29.52 ± 0.58, E = 29.33 ± 0.60, S = 28.48 ± 0.42 and ES = 26.59 ± 0.40*.*P<0.0005 vs C and E.  For euthanasia, the animals were previously anesthetized using intraperitoneal injection of ketamine hydrochloride (60 mg / kg body weight) and xylazine hydrochloride (10 mg / kg body weight) was injected after 12 h of fasting, 24 h after the last RT and smoking sessions. |
| Numbers analysed | 15 | All analyzes were performed with n = 08 per group. All animals were included in the study. |
| Outcomes and estimation | 16 | The values presented refer to the mean ± Standard Error of the Mean (SEM).  **Body mass gain (g)** – C=230.50±10.80, E=217.25±13.22, S=184.75±6.35 and ES=169.12±9.69.  **Final body weight (g)** – C= 482.62±12.71, E=463.87±15.30, S=435.12±6.80 and ES= 414.01±16.04.  **Feed consumption (g)** – **C=**29.52±0.58, E=29.33±0.60, S=28.48±0.42 and ES=26.59±0.40.  **Lee Index (**weight ^1/3^/naso-anal length in cm) – **C=**320.19±3.34, E=317.49±3.13, S=317.48±2.74 and ES=312.63±2.04.  **Skeletal muscle gastrocnemius (g)** – C=2.8±0.1, E=2.7±0.1, S=2.7±0.1 and ES=2.6±0.1.  **Goblet cell counts** (un) – C=234.12±22.93, E=259.75±30.17, S=255.25±13.26 and ES=267.87±27.63.  **Pulmonary emphysema** - Nonparametric analysis.  **Pulmonary artery thickness** (µm) – C=281.32±20.13, E=300.95±28.51, S=380.93±15.88 and ES=361.94±17.25.  **Thickness septum** **(**µm) **–** C=1287.33±109.05, E=1292.91±80.39, S=1190.61±79.24 and ES=1414.94±28.11.  **Right ventricle thickness right (**µm) –  C=663.62±41.96, E=832.61±101.72, S=1330.88±110.06 and ES=581.01±43.02.  **Left ventricle thickness leSt (**µm) – C=1524.39±14.78, E=1487.52±19.24. S=1472.08±25.66 and ES=1442.06±41.91.  **Diameter of the fibers of the gastrocnemius muscle (**µm) – C=316.49±10.60, E=330.20±12.19, S=301.15±9.86 and ES=261.61±4.15.  **Kupffer cell counting (un)** – C=443.50±47.39, E=499.00±31.69, S=595.75±28.32 and ES=619.25±19.10.  **Right ventricle Collagen Density (****Log Nr / log r-1)** **–** C=1.75±0.09, E=1.72±0.09. S=1.80±0.07 and ES=1.79±0.08.  **Left ventricle Collagen Density (Log Nr / log r-1) –** C=1.90±0.02, E=1.74±0.10, S=1.89±0.03 and ES=1.80±0.09. |
| Adverse events | 17 | 1. There were no adverse events in any experimental groups. 2. No modification in the protocol was done during all the period. |
| Interpretation/  scientific implications | 18 | 1. Although several benefits of RT have already been described in the literature, there is no consensus about its real contribution to the prevention of tissue alterations provoked by exposure to secondhand cigarette smoke. The present study aimed to evaluate the effect of resistance training on anatomopathological changes in the right and left ventricles, trachea, lung, gastrocnemius muscle and liver of rats exposed to secondhand smoke. The heart, trachea, lung, liver and gastrocnemius muscle were removed for histopathological analysis. Pulmonary emphysema (S and ES vs C and E, P <0.0001) and pulmonary artery thickness enlargement (S vs C and E, P = 0.003, ES vs C, P = 0.003) were detected in the smoking groups. There was an increase in the right ventricle thickness in the S group compared with all other groups (P <0.0001). An increase in resident macrophages in the liver was detected in both smoking groups compared with the C group (P = 0.002). Additionally, a relevant reduction of the diameter of the muscle fibers was detected only in ES compared with the C, S and E groups (P = 0.0002), impairing, at least in part, the muscle mass in exercised smoking rats. Therefore, it was concluded that resistance training prevented the increase of thickness of the right ventricle in rats exposed to secondhand cigarette smoke, but it may be not so beneficial for the skeletal muscle of smoking rats.   The present study followed the principle of 3Rs, using the least number of rats to reach statistical significance. However, we suggest future studies with some analyzes that complement our results.   1. We did not perform right cardiac catheterization (RCC) and transthoracic echocardiography (ET), which are relevant tools for the detection and screening of Pulmonary Hypertension (PH). Further studies can be developed with the intention of verifying whether resistance training prevents the development of PH using CCD and ET techniques.   Further studies characterizing the type of macrophages present in the liver and analyzing the ROS reactive oxygen species in smokers could contribute to a better understanding of the mechanisms involved in resistance training effects in this model.     1. **Replacement**   Wistar rats were used as animal model for our study since they present many similarity with humans considering physiologycal functions and they belong to a strain widely used in scientific studies. Also, as we performed the evaluation in different organs such as heart, lungs, liver, skeletal muscle, we really needed to use animal model. Due to ethical conditions it is not allowed to collect such organs of human patients.  Another important point is that the study could not be carried out in vitro or in silico since we want to investigate the effect of resistance training considering its action on the body as a whole. We chose to use male rats because of fewer hormone interference compared to female rats, besides many studies in the literature use male rats as animal model.  **Reduction**  Power analysis has been applied to reduce the number of animal used  **Refinement**  Acclimatization and pre-conditioning period  Proper handling by trained and skilled personnel  Good housing conditions: 04 animals per cage to avoid changes in specific-specie behaviour  Proper randomization and blinding procedures  Adequate exercise after MSL test.  Proper use of anaesthetics  Proper euthanasia method  Humane endpoints: signs of severe distress and pain; systemic inflammation; weight loss superior to 20% of body weight |
| Generalisability/  translation | 19 | The practice of resistance training in Chronic Obstructive Pulmonary Disease (COPD) patients and smokers, has already been investigated in humans, most of the time with qualitative methodologies, such as questionnaires. Anatomopathological observations in these risk groups are very important, after all, with these results it is possible to state more positively the positive and negative points of the practice. It is known that it is difficult to obtain human tissues for anatomopathological analysis, and that Wistar species presents similar responses to those of humans. Thus, the present study presents results that can enrich the best type of training and intensity for this population, in addition to raising hypotheses for future studies. |
| Funding | 20 | No financial support was obtained for this study. |
