# Supplementary material for: Resistance training prevents right ventricle hypertrophy in rats exposed to secondhand cigarette smoke
Source: PLoS One. 2020 Aug 7;15(8):e0236988. doi: 10.1371/journal.pone.0236988 (PMC7413484; doi:10.1371/journal.pone.0236988)
Supplement: Attachment — Submitted filename: Response.docx [file pone.0236988.s005.docx]

Firstly, I would like to thank for the suggestions.

As requested, we are presenting a new version of the manuscript.

1) The brand name and source of the cigarettes used in this study.

R: As it was a commercial cigarette, the brand of the smoke cigarette was not added, avoiding any conflict of interest, but more details about cigarette was described in the section Material and Methods. A reference (21) containing a study with similar cigarette was added in the text. (p6, lines 137-139).

2) A reference that supports the use of the maximum supported load test in rats.

R: Two references were added in the text. Ref number 23 and 24.

3) A table reporting the results of the exercise/resistance training, including the exercise capacity and MSL weight for all groups at the four different resistance training sessions.

R: A Table was added in the manuscript, p7,detailing exercise capacity and MSL weight for all groups in the beginning of the training protocol when MSL test was performed.

4) Details regarding the frequency of animal monitoring and how animals were assessed for signs of distress.

R: Some details were added in the text of the manuscript (p5, lines99-103) about animal monitoring and care to avoid distress.

5) Our editorial staff has assessed your submission, and we have significant concerns about the grammar, usage, and overall readability of the manuscript. We believe that the manuscript is much more likely to be accepted if it is easy for the reviewers to read and understand. We therefore request that you revise the text to fix the grammatical errors and improve the overall readability of the text before we send it for review.

R: The text was all reviewed and the Editing certificate by AJE website is in attachment.

Sincerely,

Dr Patricia Seraphim
