# Supplementary material for: Resistance training prevents right ventricle hypertrophy in rats exposed to secondhand cigarette smoke
Source: PLoS One. 2020 Aug 7;15(8):e0236988. doi: 10.1371/journal.pone.0236988 (PMC7413484; doi:10.1371/journal.pone.0236988)
Supplement: Attachment — Submitted filename: Response to reviewers.doc [file pone.0236988.s006.doc]

Dear Stanton A. Glantz,

Firstly, we would like to thank for the appreciattion and comments in oour paper titled “Resistance training prevents right ventricle hypertrophy in rats exposed to secondhand cigarette smoke”. The review was done, and the authors accepted the suggestions as well as implemented the information needed.

The answers for the reviwers´s questions are above, as following:

**Reviewer #1:**

**1-** “The authors state in their response to previous evaluation that they have opted to not identify the commercial cigarette brand that they have used, to avoid conflict of interest. This is not acceptable; they need to identify the cigarette because not all commercial cigarettes are the same; it’s important information. There should be no concern about conflict of interest here; identification of cigarettes used in such studies is routinely done and the only potential conflict of interest that I can see is if one of the authors works for or is funded by the tobacco company in question or the tobacco industry in general; is this is the case, then it needs to be disclosed but the cigarette still needs to be identified.”

**Answer:** The commercial cigarette brand was added to the text in Material and Methods Section of the manuscript.

**2- “**There are some essential details about the smoke exposure that are missing and should be added. Please provide some measure of smoke concentrations in the exposure chamber, as either total particulate matter or respirable suspended particles (<2.5 µm) (and potentially CO if that was measured). Is the smoking system a commercial system (if so, which one) or a custom built system? I don’t entirely follow the part about the chamber used for placement of a lit cigarette; despite describing airflow to bring smoke to the rats, there’s no mention of smoke being sucked through the cigarette to generate the smoke (what puff protocol was used; e.g., puff volume, puff duration, puff frequency). If no smoke was sucked through the cigarettes, and they were simple smoldering in place such as sitting in an ashtray, then that is a different model from what is typically used for secondhand smoke and should be explained. Are the multiple cigarettes all lit simultaneously or successively? (I’m guessing it was successively one at a time, since a cigarette probably can’t last for a whole 30 minutes, but please clarify) Were they pre-equilibrated to consistent humidity levels before use, as is common practice?”

**Answer:** The inhalation system was a custom built system composed by a closed glass box (100 x 44 x 44 cm), divided in 2 different compartments: one compartiment for allocation of the cage with 04 rats to be exposed to the cigarette smoke, and the other for the allocation of the cigarettes to be burned. A compressor of 10 L/min-air was coupled to the cigarette compartment to push the smoke to the other compartment of the box. The compartment where the animal was allocated presented a hole for the exhaust of the smoke. Four cigarettes were lit and the complete combustion occurred during 10 minutes, however the rats spent 30 minutes inside this system. So, during the 10 first minutes inside the chamber, the cigarettes were burned, but in the rest of the time the rats were exposed only to the polluted air of the environment. The dose used in the smoke exposure is equivalent to 10 to 20 cigarettes for a chronic human smoker.

4) “I understand that table 1 is being provided to address concerns about the weight bearing protocol, but it looks more like Results than Methods to me.”

**Answer:** Table 1 was included as Supplementary material – see Suppl 1.

Discussion: Regarding the sentence starting on line 324: “It is known that exposure to secondhand smoke intensifies the harmful effects of the smoke due to the absence of a filter; secondhand smoke has nicotine and CO2 concentrations three times higher than smoke experienced 336 by a smoker, and it has 50 times more carcinogenic substances (6,7).” I am not aware that this is the case, although it is popularly assumed. To the best of my knowledge, the filter does not filter out very much of the material that gets deep into the airways, and the mainstream smoke inhaled by a smoker is far more concentrated than secondhand smoke. (By CO2, did they mean CO?) They cite reference 6 and 7; 6 is a meta-analysis and 7 is a study about cluster headaches and neither supports the statement. I would remove this highly questionable sentence. This in no way minimizes the importance of secondhand smoke as a major source of adverse health effects.

**Answer:** The sentence and the references were deleted of the manuscript.

**Minor comments**
**1)** While the nomenclature of SC, EC, SS, and ES makes sense, it’s REALLY hard to follow because S stands for both Sedentary and Smoker. Yes, it matters if it’s the first or second letter of the pair, but it just makes it much less intuitive to follow along for the reader. Can the authors come up with a similarly informative but more reader-friendly way of denoting the permutations?

**Answer:** The nomenclature was changed for the groups in the manuscript. Control (C) - without interventions; Exercised (E) - performed RT; Smoker (S) exposed to secondhand cigarette smoke; Exercised Smoker (ES) - exposed to secondhand smoke exposure and performed RT.

**2)** Abstract line 37: it will be much easier to follow if a comma is added after EC. Otherwise, it’s not immediately clear whether EC belongs to the first comparison or the second. Even then, it’s a confusing line to read.

**Answer:** The sentence was changed.

**3)** The sentence starting on abstract line 41 would be more useful if there was an indication of what the finding means (i.e., tell the reader if reduction of the muscle fiber diameter good or bad).

**Answer:** The sentence was changed.

**4)** I rarely comment on keywords, but it seems to me that including “tobacco” as a keyword would be useful.

**Answer:** The word was included as keyword.

**5)** Line 57: It’s not accurate to say that the “consumption” of a cigarette is divided into particulate and gas phases; consumption is the verb. Better to say cigarette smoke consists of particulate and gas phases. Actually, it may be a language issue, but the entire paragraph is problematic, and references 6 and 7 seem like odd choices to use here. Are the authors attempting to describe the mainstream smoke that is inhaled through the filter and the sidestream smoke that is generated (not “exhaled”) by the burning tip? This is all irrelevant to gas or particle phases; the phrase on line 58 that the gas phase consists of two types of smoke does not make sense. I suggest that they replace this paragraph (and get rid of those two references) with a simple statement that the smoke inhaled by smokers is mainstream smoke, the smoke generated by the burning tip is sidestream smoke, the secondhand smoke in the air around smokers consists of a mixture of mostly sidestream smoke with some exhaled mainstream smoke, and that secondhand smoke contains high concentrations of noxious components.

**Answer:** The sentence was substitute by “Secondhand smoke is the combination of two smokes: the mainstream exhaled by smokers plus the burning end of a cigarette (sidestream). It smoke contains more noxious substances, with hundreds toxic, and some can trigger câncer”

**6)** Line 71: In “…it is fundamental to find tools…”, fundamental should probably be replaced by something like crucial or important.

**Answer:** The word “fundamental” was substituted for “crucial”, as suggested.
 **7)** Line 74: “pulmonary pressure”: do they mean pulmonary arterial pressure or increased pressure in the lungs?

**Answer:** The correct is pulmonary arterial pressure.

**8)** Line 80: I suggest clarifying the first time they say “resistance training” to make it more obvious to readers who aren’t familiar with the topic, especially given that “insulin resistance” shows up a few words later. Perhaps “Recently, exercise in the form of resistance training (RT) was classified…”

**Answer:** The sentence was changed, as suggested.

**9)** In Table 1 in the Methods; please add units (presumably g).

**Answer:** The Table 1 was deleted of the manuscript.

**10)** Line 237, I think “tricomium” is supposed to be “trichrome”.

**Answer:** The word was changed.

**11)** Line 257: what is a “Lee index”? Please clarify in the text. It’s in the figure legend but the reader has not yet encountered that when the term is first used.

**Answer:** The term was explained in details in Methods section (section 2.5).

**12)** Line 325: Cigarette smoking is not a drug; cigarettes are the drug.

**Answer:** The sentence was changed.

**13)** Line 414: “strength” is misspelled.

**Answer:** The word was corrected.

**14)** In Figure 1, the word “cigarettes” is misspelled twice, and it is missing a space in “1x day(4 days)”

**Answer:** The figure was corrected.

**15)** The legends for Figures 2-6 include magnification denotations of the form “100x” etc., which are meaningless unless the final print size of the figure is known and not useful when viewing on a monitor. Scale bars are better, and the figures actually have scale bars already but they are much too small to be seen, so it will be a simple matter to make standard visible scale bars based on them. If a magnification number is desired, please state as “10x objective” etc.

**Answer:** The legends of the figures were changed.

**16)** In figures 2-5, where quartets of histology panels are shown, it will be much simpler for the reader if panels are labeled as ES, SS, etc. rather than a, b, etc.; it saves the reader from having to look at the legend to understand the figure.

**Answer:** The labeled was changed for the abbreviation of the groups.

**17)** I suggest making the asterisks in the bar graphs larger; they are tiny and look like lint on the computer monitor.

**Answer:** The asterisks are larger.

**Reviewer #2:** The manuscript by Seraphim et al. aims at determining the effect of resistance training on various parameters in rats exposed to second hand cigarette smoke. Their main conclusion is that the training prevents right ventricle hypertrophy. They provided data that are somehow related to development of right ventricle hypertrophy such as pulmonary artery thickness and lungs histopathology, as well as some supportive information such as body mass gain, food consumption, and masses of the different tissues that they included in their study. Not being an expert on the field, this reviewer cannot say how novel and interesting the findings are for the scientific community. However, there are definitely a number of issues that have to be addressed before the manuscript can be considered for publication.

**General observations:**
**1)** This reviewer was invited as an animal experiment ethics specialist. In this respect, the study is fine; there seems to be no ethical concern.

**2)** Although a language correction has been performed on the manuscript, clarity of the text is still below publishable quality. To illustrate it, this reviewer performed editing of a 1.5 pages section of Materials and Methods. a/ line 120-122: “The smoking rats (SS and ES) were exposed to secondhand cigarette smoke (19) for 16 weeks, which was predetermined to characterize long-term exposure (20).” The second part of the sentence may mean that 16 weeks of exposure is considered as a long-term one.

**Answer:** Sixty weeks of exposure means long-term exposure, considering rats can llive during 24 months.

According the literature (<https://doi.org/10.1590/S0102-67202012000100011>) each rat month in adulthood is similar to around 2.5 human years.

#### So we have:

#### 1 month in adulthood rat corresponding to 2.5 human years and

#### 4 months (16 weeks) in adulthood rat corresponding to around 10 human years,

#### So, 16 week smoke exposure should be considered long-term exposure.

Anyway, the sentence was removed from the text, stopping at 16 weeks in the paragraph, as following:

“The smoking rats (S and ES) were exposed to secondhand cigarette smoke for 16 weeks”.

**b/** line 125: Change “characterizing” to “representing”.

**Answer:** The sentence was rewritten.

**c/** line 138: Cigarettes never contain carbon monoxide, which is a gas. The indicated 10 mg should be the average dose a smoker receive during a smoke.

**Answer:** The sentence was changed for: “Commercial cigarettes were used in the study, containing 10mg of tar, 0.8 mg of nicotine, and 10 mg of carbon monoxide during the combustion”.

d/ line 153-154: “This test was performed only in this moment.” ???? Better delete.
**Answer:** The sentenced was deleted.

**Specific comments:**

**1)** The overall design of the study indicates that more results have been anticipated than have actually been resulted. That creates some problems. The organ masses were properly presented as not significantly altered in line 260. However, according to PLOS ONE’s publishing policies “data not shown” is not permissible. Either the data should be presented, or the observation should be omitted. In this case, I suggest presenting the data as a supplementary table.

**Answer:** The Table with the organ masses is presented as supplementary material.

**2)** line 271: On what basis was pulmonary emphysema diagnosed? Put it in the Methods section.

**Answer:** The sentence was added in Methods section: “Pulmonary emphysema was diagnosed when the alveolar spaces were enlarged and the alveolar septa retracted”.

**3)** Fig 4: Exercise increased right ventricle thickness in non-smoker rats while decreased it to BELOW THE CONTROL LEVEL in smoker ones. These data are very hard to understand.

**Answer:** In fact, **smoking** caused increase of the right ventricle thickness (see S group). Resistive training did not change the thickness of left and right ventricles nor in non-smoking (E) nor in smoking (ES) rats.

4. The number of Kupffer cells in the liver is presented in Fig 6. Either move this figure to the supplementary section or explain in the Discussion section the relevance of the data presented in it to right ventricle hypertrophy.

**Answer:** The paragraph was changed correlating liver and heart diseases and the figure 6 was maintained in the manuscript.

5. The Discussion section is poor. Please, interpret the data presented in the Results section regarding right ventricle hypertrophy and related studies on the field. Also, please avoid drawing conclusions on unsupported data.

**Answer:** Thanks for the suggestion, changes were included in the Discussion Section, as a trying to follow the suggested.
