# Supplementary material for: Resistance training prevents right ventricle hypertrophy in rats exposed to secondhand cigarette smoke
Source: PLoS One. 2020 Aug 7;15(8):e0236988. doi: 10.1371/journal.pone.0236988 (PMC7413484; doi:10.1371/journal.pone.0236988)
Supplement: Attachment — Submitted filename: Response to reviewers.doc [file pone.0236988.s007.doc]

**Response to reviewers**

We would like to start by acknowledging the thoroughness of the revision and the importance of the reviewer’s comments that greatly improved our manuscript.

The answers for the reviewers’ questions are below, as following:

**Reviewer #1:**

**Comment 1)** First of all, an important note: there are two versions of the revised manuscript, one clean and the other with changes tracked, but the two version are not identical. I realized in the middle of reviewing that at least one change made in the tracked changes version is not changed in the untracked version (see SS in line 304 of the clean version that has been changed to S (with the line through the deleted extra S) in corresponding line 314 of the tracked changes version. I don’t know if more slipped through, but it means that the tracked changes version is the one that should be used, or else some changes might become unchanged.
**Response to comment 1 -**: I would like to apologize because the final file of the Manuscript with untracked changes was really different of the version with tracked changes. I have made a mistake during the submission process, changing the files. I apologize about this mistake. But I am sure that this error did not compromise the relevance of the manuscript and the comprehension of the text in the manuscript.

**Comment 2)** The authors have made various changes to respond to previous critiques. I think the new nomenclature for the four groups is much clearer than the previous one; a good solution. Note that the changes in group designations all show up in the changes tracked version but as mentioned above, at least one of them is not in the untracked version.

**Answer to comment 2 –** We agreed with the reviewer. The designation of the groups was much better. This time, we took care about using the same designation for all groups in the untracked version. We have checked all nomenclature before sending the new version.

**Comment 3)** **A)** One remaining issue that we still need to fix is the lack of clarity about the smoke exposure system, despite the authors having added more details. The problem is that with a custom-built system, details of how the system works are important so that the results can be put in context of other smoke exposure literature. From the new description added, it’s still not clear to me how the smoke is being generated, and they refer to one of their previous papers but it is in Portuguese. Another new reference is to a review article about various effects of smoke on the lung; there’s no procedural detail there that is relevant to this paper. Most studies that produce secondhand smoke follow some variation of a pretty consistent workflow: air is sucked through a cigarette by some mechanism and according to a specific puff protocol (e.g. ISSO, Health Canada Intense), and the smoke from the burning tip is collected. Cigarettes are frequently pre-equilibrated to consistent humidity before use. These conditions provide not just consistency between experiments, but also the ability to place the results in context of the rest of the body of smoke research.

**3 B)** For example, there’s no mention in the description of air going through the cigarettes; just that the cigarettes are in a compartment and air goes from that compartment to the rats. Does that mean the cigarettes are sitting on the floor of the compartment simply smoldering in place, with no periodic puffing? If so, it would not invalidate the system, but it also would not be consistent with how such studies are typically accomplished and that should be specifically mentioned. The chemistry is different if the cigarette is smoldering in an ashtray for 10 minutes vs. if someone is actually smoking it and puffing occasionally. Please show a photograph or a diagram, and make the air path clear.

**3 C)** It’s also not clear to me what they mean when they say that the dose of smoke exposure is equivalent to 10 to 20 cigarettes for a chronic human smoker, because their model is secondhand exposure; that can’t be directly compared with the number of cigarettes being smoked by people.

**Answer to comment 3 - A)** The reference 20 in Portuguese was changed for another reference published in English by the same group: Garcia BC, Bonfim MR, Camargo RCT, Souza DRS, Abreu LC, Filho, JCSC. Effects of passive smoking associated with physical exercise in the skeletal muscle of rats during pregnancy and lactation. Int J Morphol. 2015;33:497-506. This study is from a research group that used the same chamber of exposure. We also added the reference # 18 in the line 138 (tracked version) or line 130 (untracked version), to justify the use of the model of chamber of exposure to cigarette smoke. But, in both articles, there is no adittional detail how the air flow was generated and the direction of this flow. As it is a custom-built system, we agreed with the reviewer, and more details are crucial to understand how the system works. In this new version we are adding the Fig. 1 showing the detail of the air flow inside the chamber and in the text we are describing the system more detaily. Because of this, we had to change the number of all figures in the manuscript as well.

**Answer to comment 3 - B)** During the exposure, the cigarettes were lighted and were left burning with no periodic puffing. Cigarettes were not pre-equilibrated to consistent humidity before the use. But 4 animals per time were let in the exposure chamber. And in the next day, we changed the sequence of the cages that would begin the exposure. A more detailed description was added in the Methods (lines 131-144 in the untracked version).

**Answer to comment 3 - C)** We agreed with the reviewer when he said that the dose of smoke exposure can’t be directly compared with the number of cigarettes being smoked by people, because of the model is secondhand exposure. The sentence was withdrawn (see lines 131-133 in the tracked manuscript).

**Comment 4)** The other changes have all been made satisfactorily. I noted a misspelling in the newly added material, “simoustaneouly” in the second-to-last paragraph of the discussion (line 460 in the tracked changed version).
**Answer to comment 4 –** The word was corrected to simultaneously (line 432).

**Reviewer #2:** **Comment 1)** Although the English of the manuscript has improved (at least the first 15 pages), it is still far from tolerable (at least by this reviewer). The manuscript either has not been revised grammatically, or the proof-reader has made a very poor job. This reviewer could not evaluate the revised manuscript because of the quality of English. Proof-reading is outside the reviewers' responsibilities, although the other reviewer very kindly has also suggested a number of such mistakes in the first round. In an original submission, the mistakes could be tolerated, however, not in a revised manuscript. This reviewer has got as far as the top of page 16 before quitting, and does not want to waste his/her time on submissions that do not reflect due care from the authors' side.

**Answer: -**: We would like to apologize about the grammar mistakes. We have sent the certificate of the English review made in the first version of the manuscript in attachment. After this, we added some new sentences in the manuscript to answer the reviewers, and to adequate the manuscript. However, we did not submit under an English review again, but we believe in that our inclusion in the text did not compromise the relevance of the study. We really believe in our results in this manuscript. We really believe in that the manuscript is adequate now.

**Comment 2)** **-** page 14, line81-83: Clarify the sentence. Most probably the first comma is unnecessary. Regardless, as presented, the third part of the sentence does not confer comprehensible meaning. We really believe in the manuscript is adequate now.

**Answer:** The third part of the setence explains the difference between anaerobic and aerobic training. If the pauses of recovery among the series were not done, the exercise would become aerobic. High-intensity and short-term duration with pauses among series characterize anaerobic exercise. The sentence was modified for better comprehension (see lines 82-84 in the both untracked and tracked versions).

**Comment 3)** **-**page 14, line 98: Change “monitoring” to “monitored”. Change “intake food” to “intake of food”.

**Answer:** all changes were done.

**Comment 4)** **-**page 15, line 120: Change “04” to “4”, then do the same throughout the text.

**Answer:** all changes were done

**Comment 5)** **-**page 15, line 121: Delete “only”.

**Answer:** all changes were done.
